# Emerging technologies for diagnostic pathology

**DOI:** 10.15190/d.2015.38

**Published:** 2015-06-30

**Authors:** Octavian Bucur

**Affiliations:** Department of Pathology, Harvard Medical School and Beth Israel Deaconess Medical Center, Boston, MA, USA; Institute of Biochemistry of the Romanian Academy, Bucharest, Romania

**Keywords:** Emerging technologies, methods, diagnosis, FISSEQ, expansion microscopy, 3D microscopy, 3D diagnosis, image analysis, computational pathology

## Abstract

Many technological advances have been made in the recent years, several of them with a great potential of significantly improving the diagnostic pathology field. This article discusses three of the most promising technologies, which emerged in the last one year. Fluorescent in situ sequencing can lead to the simultaneous identification of the transcriptome-wide RNA in individual cells across a tissue sections. 3D microscopy together with advanced image analysis can be used in diagnostic pathology and will especially be useful in hard to diagnose cases where the spatial relationship of the tissue components is important. Expansion microscopy physically expands the biological specimen, and is of great interest for diagnostic pathology since the cheap conventional microscopes can be used to image a symmetrically expanded tissue. In addition, digital analysis and computational pathology are an integral part of each of these three emerging technologies, which underline their importance for the future developments in diagnostic pathology.

## 1. Introduction

Several technologies with great potential in significantly advancing the diagnostic pathologies have emerged in the recent years. This article focuses on three revolutionary technologies that emerged in the last one year. These methods promise to advance the diagnostic in pathology by adding another dimension (3D diagnosis), sequencing in situ (fluorescent in situ sequencing) and tissue expansion (expansion microscopy). These technologies combined with automated pathology have the potential of being widely employed by the pathologists in the near future.

## 2. Fluorescent in situ sequencing

Many approaches using high-throughput methodologies have resulted in an extraordinary development of the “omics” fields: genomics, transcriptomics, proteomics, metabolomics, interactomics, immunomics, and others^1,2^ etc. Simultaneous determination and analysis of hundreds/thousands of genes (genomics), transcripts (transcriptomics), proteins (proteomics) can provide an unprecedented insight into the organization, regulation and function of biological systems^1^.

Researchers and pathologists understand now more than ever the importance of personalized medicine. The potential impact of next-generation sequencing and whole-genome analysis in medicine and, specifically, in clinical laboratory practice is well recognized^3,4^. However, the current sequencing techniques, although high-throughput, do not provide much information on the exact location of its target. Moreover, DNA/RNA FISH identifies the exact location of the target, however, it is not a high-throughput technique^5^, since it is limited in the number of probes that can be simultaneously or consecutively employed. Identifying the exact location of the sequenced or identified RNA/DNA of interest on the tissue section would bring an enormous benefit not only to the research community, but also to the diagnostic pathology field. Fluorescence in situ sequencing (FISSEQ) is a highly multiplexed subcellular RNA sequencing *in situ*, which permits massively parallel detection of genetic elements, including gene transcripts, important in analyzing cellular phenotypes, gene regulation, and cellular environment in situ^6,7^. This transcriptome-wide RNA sequencing in situ was validated on multiple specimen types and spatial scales. Fisseq is compatible with fresh frozen brain tissue sections and whole-mount embryos, although the authors only present the complete sequencing of cell lines^6^.

Briefly, the cells/tissue are fixed and the RNA is revers transcribed into cDNA using random hexamer primers. cDNA is then *in situ *amplified and cross-linked, forming the single-stranded DNA nanoballs (also named rolonies or cDNA amplicons). Fluorescent sequencing, which consists in using a fluorescent probe hybridized to the adaptor sequence and imaged by confocal microscopy, is next performed on the DNA nanoballs^6,7^.

If successful on tissue sections, this method would enable the simultaneous identification of the transcriptome-wide RNA in individual cells across a tissue sections. This will not only help scientists to study *in situ* cellular interactions (such as the ones from epithelium and stroma in several types of malignancies (breast, gastro-intestinal tumors etc)), immune cells and cancer associated fibrobalsts/tumor cells etc) but will also help in diagnosis and post-diagnosis follow up of many pathologies, including cancers.

## 3. 3D diagnosis in pathology

Since the early 20^th^ century, pathological classification of many types of malignancies has been primarily based on the visual analysis of hematoxylin and eosin stained image slides using conventional two dimensional (2D) microscopy^8^. With the recent development of new state-of-the-art microscopy platforms, such as the fluorescent *lightsheet microscopy* (the Nature Methods’ method of the year 2014)^9-11^ and *multiphoton microscopy (2015 Brain Prize given for the development of 2 photon microscopy)^12^*, the rapid acquisition of three dimensional (3D) images directly from tissue samples up to several millimeters in thickness is now possible^11^.

Recent results presented a successful pipeline of formalin fixed paraffin embedded tissue sample preparation, imaging with a 3D advanced microscopy and extraction of quantitative image measurements from the 3D images to build classification models in breast tissue. This classification models help in differentiating normal tissue from non invasive breast cancer and different grades of invasive breast cancer^13,14^.

Thus, 3D microscopy together with advanced image analysis can be used in diagnostic pathology and will be especially useful in hard to diagnose cases where the spatial relationship of the tissue components is important.

## 4. Expansion microscopy

Researchers have successfully created state of the art microscopes, which are under continuous development, in order to provide high resolution images and to see in details the structures of the different types of cells and tissue^9-12^. However, a revolutionary idea recently came to life when a 4-5 fold expansion of the human brain tissue was reported with minimal distortion^15^. The authors named this method, of physical magnification of the specimen itself, expansion microscopy (ExpM)^15^. ExpM would be a great asset in diagnostic pathology, since one could use a cheap conventional microscope to image a symmetrically expanded tissue.

ExpM uses a “swellable” polymer network in a biological sample that can expand the sample by 4-5 fold^15,16^. The method uses the well known properties of the polyelectrolyte gels, which after water addition they expand. The tissue is placed in a polyelectrolyte gel, followed by its treatment with a protease to homogenize its mechanical characteristics and the placement of the specimen in water for expansion^15^.

Expanded tissues (such as the brain) have the great advantage that the process makes them completely or partially transparent. Thus, these tissues are well fit for lightsheet microscopy, which requires transparent specimens, so there is no need for a preclearing step^17^.

**In conclusion**, these are only few of the several important technological advances that could help the diagnostic pathology field in the near future. Although these methods are still under development, they have an extraordinary potential in transforming diagnosis of different pathologies. Moreover, these three techniques can be combined together and employed for analysis of the same samples, leading to valuable results for both research and diagnosis.

## References

[1] Finn William G (2007). Diagnostic pathology and laboratory medicine in the age of "omics": a paper from the 2006 William Beaumont Hospital Symposium on Molecular Pathology.. The Journal of molecular diagnostics : JMD.

[2] Wang Xiaoju, Yu Jianjun, Sreekumar Arun, Varambally Sooryanarayana, Shen Ronglai, Giacherio Donald, Mehra Rohit, Montie James E, Pienta Kenneth J, Sanda Martin G, Kantoff Philip W, Rubin Mark A, Wei John T, Ghosh Debashis, Chinnaiyan Arul M (2005). Autoantibody signatures in prostate cancer.. The New England journal of medicine.

[3] Tonellato Peter J, Crawford James M, Boguski Mark S, Saffitz Jeffrey E (2011). A national agenda for the future of pathology in personalized medicine: report of the proceedings of a meeting at the Banbury Conference Center on genome-era pathology, precision diagnostics, and preemptive care: a stakeholder summit.. American journal of clinical pathology.

[4] Haspel Richard L, Saffitz Jeffrey E (2014). Genomic oncology education: an urgent need, a new approach.. Cancer journal (Sudbury, Mass.).

[5] Ginart Paul, Raj Arjun (2014). RNA sequencing in situ.. Nature biotechnology.

[6] Lee Je Hyuk, Daugharthy Evan R, Scheiman Jonathan, Kalhor Reza, Yang Joyce L, Ferrante Thomas C, Terry Richard, Jeanty Sauveur S F, Li Chao, Amamoto Ryoji, Peters Derek T, Turczyk Brian M, Marblestone Adam H, Inverso Samuel A, Bernard Amy, Mali Prashant, Rios Xavier, Aach John, Church George M (2014). Highly multiplexed subcellular RNA sequencing in situ.. Science (New York, N.Y.).

[7] Lee Je Hyuk, Daugharthy Evan R, Scheiman Jonathan, Kalhor Reza, Ferrante Thomas C, Terry Richard, Turczyk Brian M, Yang Joyce L, Lee Ho Suk, Aach John, Zhang Kun, Church George M (2015). Fluorescent in situ sequencing (FISSEQ) of RNA for gene expression profiling in intact cells and tissues.. Nature protocols.

[8] Beck Andrew H, Sangoi Ankur R, Leung Samuel, Marinelli Robert J, Nielsen Torsten O, van de Vijver Marc J, West Robert B, van de Rijn Matt, Koller Daphne (2011). Systematic analysis of breast cancer morphology uncovers stromal features associated with survival.. Science translational medicine.

[9] Keller Philipp J, Schmidt Annette D, Wittbrodt Joachim, Stelzer Ernst H K (2008). Reconstruction of zebrafish early embryonic development by scanned light sheet microscopy.. Science (New York, N.Y.).

[10] Keller Philipp J (2013). Imaging morphogenesis: technological advances and biological insights.. Science (New York, N.Y.).

[11] (2015). Method of the Year 2014.. Nature methods.

[12] (2015). Prize Winners 2015; http://www.thebrainprize.org/flx/prize_winners/previous_prize_winners/. TheBrainPrize.

[13] (2015). http://www.aacr.org/Documents/15AM_SITawardeesforProgram.pdf. AACR Awards.

[14] Bucur Octavian, Irshad Humayun, Montaser-Kouhsari Laleh, Knoblauch Nicholas W., Oh Eun-Yeong, Nowak Jonathan, Beck Andrew H. (2015). Abstract 3477: 3D morphological hallmarks of breast carcinogenesis: Diagnosis of non-invasive and invasive breast cancer with Lightsheet microscopy. Cancer Research.

[15] Chen Fei, Tillberg Paul W, Boyden Edward S (2015). Optical imaging. Expansion microscopy.. Science (New York, N.Y.).

[16] Strack Rita (2015). Bigger is better for super-resolution.. Nature methods.

[17] Dodt Hans-Ulrich (2015). Microscopy. The superresolved brain.. Science (New York, N.Y.).

